# Does oxytocin lead to emotional interference during a working memory paradigm?

**DOI:** 10.1007/s00213-017-4737-z

**Published:** 2017-09-14

**Authors:** Marieke S. Tollenaar, M. Ruissen, B. M. Elzinga, E. R. A. de Bruijn

**Affiliations:** Institute of Psychology, Department of Clinical Psychology, Leiden University and Leiden Institute for Brain and Cognition, PO Box 9555, 2300 RB Leiden, The Netherlands

**Keywords:** Oxytocin, Working memory, Emotion, Interference, Distractor, Childhood maltreatment

## Abstract

**Background:**

Oxytocin administration may increase attention to emotional information. We hypothesized that this augmented emotional processing might in turn lead to interference on concurrent cognitive tasks. To test this hypothesis, we examined whether oxytocin administration would lead to heightened emotional interference during a working memory paradigm. Additionally, moderating effects of childhood maltreatment were explored.

**Methods:**

Seventy-eight healthy males received 24 IU of intranasal oxytocin or placebo in a randomized placebo-controlled double-blind between-subjects study. A working memory task was performed during which neutral, positive, and negative distractors were presented.

**Results:**

The main outcome observed was that oxytocin did not enhance interference by emotional information during the working memory task. There was a non-significant trend for oxytocin to slow down performance irrespective of distractor valence, while accuracy was unaffected. Exploratory analyses showed that childhood maltreatment was related to lower overall accuracy, but in the placebo condition only. However, the maltreated group sample size was very small precluding any conclusions on its moderating effect.

**Conclusions:**

Despite oxytocin’s previously proposed role in enhanced emotional processing, no proof was found that this would lead to reduced performance on a concurrent cognitive task. The routes by which oxytocin exerts its effects on cognitive and social-emotional processes remain to be fully elucidated.

## Introduction

Oxytocin research has received much attention in recent years due to its positive role in social behaviors including pair bonding, trust, social memory, and anxiety (Heinrichs et al. 2009; Macdonald and Macdonald 2010; Meyer-Lindenberg et al. 2011). These effects have raised the possibility of a host of therapeutic applications (Guastella et al. 2009a; Koch et al. 2014; McQuaid et al. 2014). Oxytocin administration has been hypothesized to increase socially oriented or approach-related behaviors (Harari-Dahan and Bernstein 2014; Kemp and Guastella 2010; Radke et al. 2013; Shamay-Tsoory and Abu-Akel 2016), which may be due to increased salience of emotions and/or enhanced emotion recognition (Shahrestani et al. 2013; Van IJzendoorn and Bakermans-Kranenburg 2012). However, while oxytocin is often assumed to have positive, pro-social effects, recent findings also indicate that under certain circumstances oxytocin administration can have less beneficial or even anti-social effects, such as enhanced aggression, anxiety, or distrust (De Dreu et al. 2010; MacDonald et al. 2013; Radke and de Bruijn 2012). This may depend on contextual and personal factors (Bartz et al. 2011; Carter 2014; Harari-Dahan and Bernstein 2014). Clearly, before oxytocin administration can be introduced as a possible new therapy in clinical practice, its working mechanisms and potential moderators should be carefully studied.

While many studies have focused on the effects of oxytocin on complex social behaviors, recent evidence indicates that oxytocin may already act on early emotional attention processes (Domes et al. 2013; Ellenbogen et al. 2012, 2013; Prehn et al. 2013; Ruissen and de Bruijn 2015), which could underlie changes in more complex social behaviors. In a previous study, we showed that oxytocin administration in healthy young men led to enhanced orienting of attention in response to emotional (both happy and fearful) gaze cues (Tollenaar et al. 2013). Others have also shown that oxytocin administration may enhance the processing of social or emotional information at early, more automatic stages by enhancing detection accuracy of emotional expressions (Schulze et al. 2011), increasing recruitment of attentional resources (Prehn et al. 2013), and by directing covert attention to social cues (Domes et al. 2013). Some of these studies found the effects to be more pronounced for positive emotions (Domes et al. 2013; Schulze et al. 2011), and Ellenbogen et al. (2012) even showed an attenuated emotional bias for negative emotions. Null findings have been reported as well (Guastella et al. 2009b), showing that the effects of oxytocin may be task and valence specific. Hence, more research is needed to clarify the emotional attention effects of oxytocin.

While enhanced attention to emotional information may enhance social processes, it could potentially also interfere with concurrent cognitive processes (Cromheeke and Mueller 2014). Previous work in our group has shown that emotionally arousing information can interfere with working memory performance (Oei et al. 2010, 2012), and that this interference may be more severe in disorders characterized by emotional dysregulations (Krause-Utz et al. 2012). Increased processing of emotions due to oxytocin administration in such contexts might lead to even stronger interference of cognitive processing. A study by Ellenbogen et al. (2013) showed reduced inhibition of sad facial expressions in participants scoring high on depressive symptoms after oxytocin administration, pointing to possible interference by emotions on an automatic cognitive process. Enhanced distractibility by emotions due to oxytocin could give contraindications of oxytocin administration in patient groups, or in situations where emotional distractors are present while cognitive performance is of importance.

In the current study, we therefore examined whether intranasal oxytocin administration would enhance interference by emotional (positive and negative) information during a concurrent working memory task, which has previously been shown to be sensitive to emotional distraction (Krause-Utz et al. 2012; Oei et al. 2010, 2012). We hypothesized that intranasal oxytocin administration would enhance interference by emotional (positive and negative) information during the working memory task, as indicated by an increase in reaction times and/or errors (i.e., reduced accuracy) in a healthy male population.

Furthermore, as mentioned previously, the effects of oxytocin may depend on contextual and personal factors. One factor that could be an important moderator of the effects of oxytocin is the experience of early life stress, including childhood maltreatment. Early life stress has been related to reduced (or less beneficial) effects of oxytocin on pro-social behavior, neural and biological responses (Fan et al. 2014; Grimm et al. 2014; Van IJzendoorn et al. 2011), which could be due to early life stress-related changes in the oxytocinergic system (Heim et al. 2009b). In the current study, we therefore additionally want to explore the possible moderating effects of childhood maltreatment on the effects of oxytocin administration.

## Material and methods

### Participants

Seventy-eight Dutch healthy male volunteers participated in the study. Only males were included due to additional social paradigms included in the full study protocol (see also Ruissen and de Bruijn (2015)). Exclusion criteria were use of medication, medical or physical illness, current psychiatric problems, excessive drug or alcohol use, and excessive smoking (> 15 cigarettes per day), based on self-report. Three participants (two in the placebo and one in the oxytocin condition) were excluded from analyses, because they did not follow the instructions of the working memory task (> 50% errors). Data of the remaining 75 participants (mean age = 22.6 years, SD = 3.6, range = 18–35) were included in the analyses. All participants signed informed consent and received a payment of 20 euros for their participation.

### Procedures

A double-blind placebo-controlled between-subjects design was used. Participants were randomly assigned to either the oxytocin (*N* = 39, mean age = 22.3, SD = 3.1) or the placebo condition (*N* = 36, mean age = 22.8, SD = 4.1, *F*(1, 73) = 4.64, *p* = .55). Randomization was performed and controlled by the pharmacy of the Leiden University Medical Center (LUMC). Participants in the oxytocin condition received a nasal spray containing oxytocin (Defiante Farmaceutica, Sigma-Tau). Participants in the placebo condition received a nasal spray containing a solution of sodium chloride. Participants self-administered six puffs (three in each nostril), resulting in a total of 24 intranasal units (IU). This dose has been shown to induce both behavioral and cognitive effects (Quintana et al. 2015; Shahrestani et al. 2013). Participants came to the lab in pairs for a test session, which included a social interaction task, an EEG paradigm (Ruissen and de Bruijn 2015), and the emotional working memory task described here. The current task was performed last in the study protocol, as the emotional distractors used in this working memory paradigm can be perceived as stressful and could have influenced later tests. Hence, approximately 80 min after oxytocin/placebo administration, the emotional working memory task was performed individually. Previous studies have shown that oxytocin stays detectable in saliva up to 7 h after administration (van IJzendoorn et al. 2012), and physiological and emotional effects have been reported after similar time spans (Olff et al. 2013). Procedures were in accordance with the Declaration of Helsinki and approved by the Leiden University Medical Ethics Committee.

### Measures

#### Emotional working memory task

The EWMT is an adapted Sternberg item recognition task, which has repeatedly shown interference by emotional information on working memory performance in both healthy and clinical samples (Krause-Utz et al. 2012; Oei et al. 2010, 2012). The overall goal of this task is for participants to memorize a list of letters and then to check in a new list of letters whether they recognize one of the previously shown letters. The time interval between the two lists is used to present distracting information, in this case pictures of different valences. The current paradigm consisted of 72 trials, each starting with a black fixation block for 750 ms, followed by the presentation of three black letters below each other (memoranda, 1000 ms). After a delay interval (1500 ms), three letters were again displayed (probe, ≤ 2000 ms). In half of the trials, one of the three memoranda (target) was present in the probe. Participants had to press a “yes” or “no” button indicating whether they had recognized a target or not. Participants had to respond within the 2000 ms during which the probe was presented, which would start the next trial. During the delay interval, neutral, positively, or negatively arousing pictures from the International Affective Picture System (IAPS, Lang et al. 2008) were presented as distractors. The positive and negative pictures mainly included social scenes and human body parts, while the neutral pictures included natural scenes and objects. The 24 positive, negative, and neutral pictures were randomly displayed. The inter-trial interval was 500 ms. Participants were instructed to focus on the middle of the screen, concentrating only on the memory task and ignoring the pictures during the delay. Effects of the distractor valence on both speed and percentage errors were analyzed to examine interference.

#### Childhood Trauma Questionnaire

The Childhood Trauma Questionnaire (CTQ) short form (Bernstein et al. 2003; Thombs et al. 2009) is a 28-item retrospective self-report questionnaire developed to measure five types of childhood maltreatment. The five types are emotional, physical, and sexual abuse and emotional and physical neglect. Each scale of the CTQ encloses five items that are scored on five-point Likert scales with a maximum score of 25. The scales showed good reliability in the current sample (Cronbach’s alphas > 0.75), except for the physical neglect scale (Cronbach’s alpha = 0.50). Due to a technical error, CTQ scores were only acquired for 56 participants (75%). No differences between this group and the group without the CTQ data were found on age or task performance (all *p*s > .08). Given the healthy sample with a low prevalence of childhood maltreatment, and hence highly skewed distribution of the CTQ data, instead of continuous scores, we made use of the moderate-severe cutoff scores of the CTQ (Bernstein et al. 2003; Heim et al. 2009a), to identify cases with a positive history of childhood maltreatment (≥ 13 for emotional abuse, ≥ 10 for physical abuse, ≥ 8 for sexual abuse, ≥ 15 for emotional neglect, and ≥ 10 for physical neglect). If minimally 1 moderate to severe type of trauma was reported, maltreatment was scored as present (*n* = 16; 9 placebo, 7 oxytocin), otherwise as no (/mild) maltreatment (*n* = 40; 19 placebo, 21 oxytocin).

### Statistical analyses

Reaction times (RTs) of correct trials were checked for outliers (more than two SD above the individual mean per type and emotion), which were then replaced by the individual mean ± two SD per type and emotion (4.4% of the total trials). Mean RTs of correct trials and the percentage of errors were analyzed using repeated measures (RM) analyses of variance (ANOVA), with the within-subject factors emotion (neutral, negative, and positive) and trial type (target present or absent) and the between-subjects factor condition (oxytocin or placebo) in the full sample (*N* = 75). For exploratory analyses, the factor maltreatment was included as a between-subjects factor within the subset of individuals with these data available (*n* = 56). The Greenhouse-Geisser correction was applied, if the sphericity assumption was not met. Effect sizes (partial *η*_p_^2^) are reported. The threshold for statistical significance was set at *p* < .05 (two tailed).

## Results

### Reaction times

Table 1 shows reaction times for the correct trials with either a target present or a target absent after neutral, negative, and positive distractors in the placebo and oxytocin group. As expected, the RM analyses on RTs in the full sample indicated interference by both negative and positive distractors. That is, participants were slower on trials with negative and positive distractors (mean RTs = 1098 and 1060 ms, SDs = 16.8 and 17.4, respectively) compared to neutral images (mean RTs = 1008 ms, SD = 17.5; main effect of emotion *F*(2, 146) = 41.42, *p* = < .001, *η*_p_^2^ = 0.36, post hoc comparisons *p*s < .001). Response times in target absent trials were slower compared to target present trials (*F*(1, 73) = 68, 26, *p* < .001 *η*_p_^2^ = 0.48), and the effect of the emotional distractors was stronger in the target absent trials (emotion by trial type interaction *F*(2, 146) = 6.07, *p* = .003, *η*_p_^2^ = 0.077).

**Table 1:** Mean RTs (SD) in milliseconds and percentage of errors (SD) on target present and target absent trials within the placebo and oxytocin group

Condition:	Placebo (*N* = 36)			Oxytocin (*N* = 39)		
Distractor:	Neutral	Negative	Positive	Neutral	Negative	Positive
RT target present trials:	932 (127)	1014 (148)	948 (136)	995 (180)	1104 (172)	1034 (167)
RT target absent trials:	1037 (175)	1108 (175)	1109 (182)	1069 (169)	1167 (157)	1151 (181)
% Error target present trials:	21.3 (10.4)	27.8 (10.9)	20.8 (11.5)	20.5 (15.5)	28.8 (14.0)	21.2 (10.4)
% Error target absent trials:	7.4 (9.9)	22.7 (11.0)	7.6 (8.5)	4.5 (8.1)	24.6 (9.9)	8.3 (9.6)

**Fig. 1:**
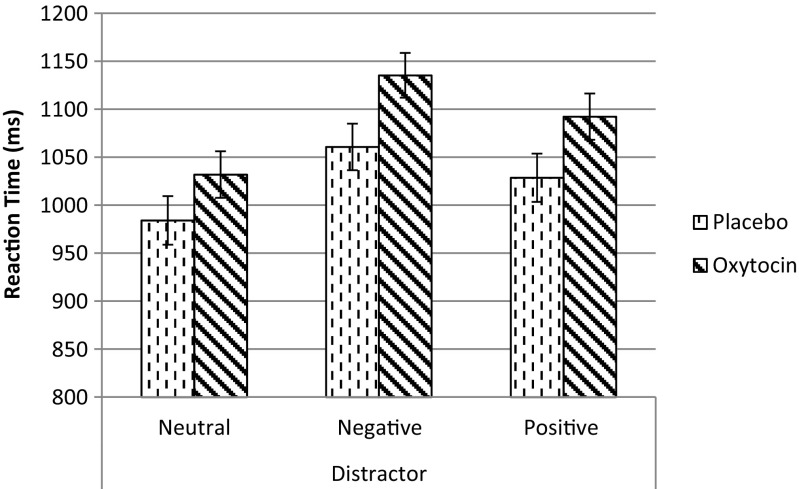
Reaction times (ms) for correct trials per emotional distractor on the working memory task in the placebo (*n* = 36) and oxytocin condition (*n* = 39). Error bars represent standard errors

There was no significant main effect of condition, although a trend was apparent (*F*(1, 73) = 3.64, *p* = .060, *η*_p_^2^ = 0.047), indicating marginally slower reaction times in the oxytocin condition (mean RT = 1087 ms, SD = 22.5) compared to placebo (mean RT = 1025 ms, SD = 23.5). However, no evidence was found for enhanced distraction by emotional information after oxytocin administration, as the interaction between condition and emotion was not significant (*F*(2, 146) = 0.92, *p* = .40, *η*_p_^2^ = 0.012), see Fig. 1.

### Errors

Table 1 also shows the percentage of errors after neutral, negative, and positive distractors on target present and target absent trials in the placebo and oxytocin group. The RM analyses on the percentage of errors in the full sample indicated interference by negative distractors, but not by positive distractors. That is, participants made more errors on trials with negative distractors (mean percentage error = 26.0, SD = 1.0) compared to neutral images (mean percentage error = 13.4, SD = 1.0, *F*(1, 73) = 135.4, *p* < .001, *η*_p_^2^ = 0.650), but not on trials with positive distractors (mean percentage error = 14.5, SD = 0.9, *F*(1, 73) = 1.07, *p* = .30, *η*_p_^2^ = 0.014; main effect of emotion *F*(2, 146) = 87.11, *p* < .001, *η*_p_^2^ = 0.544). Furthermore, participants made more errors in the target present trials compared to target absent trials (main effect of trial type *F*(1, 73) = 114.11, *p* < .001, *η*_p_^2^ = 0.61), and the effect of the emotional distractors was stronger in the target absent trials (emotion by trial type interaction *F*(2, 146) = 11.71, *p* < .001, *η*_p_^2^ = 0.138).

There was no main effect of condition (*F*(1, 73) = .001, *p* = .98, *η*_p_^2^ < 0.001). Again, no evidence was found for an enhanced distraction by emotional information after oxytocin administration, as the interaction between condition and emotion was not significant (*F*(2, 146) = 1.32, *p* = .27, *η*_p_^2^ = 0.018).

### Childhood maltreatment as a moderator

When childhood maltreatment history was added as a factor to the RM analyses on RTs, there was no significant main effect of condition (*F*(1, 52) = 2.01, *p* = .16, *η*_p_^2^ = 0.037), or of maltreatment history (*F*(1, 52) = 0.23, *p* = .64, *η*_p_^2^ = 0.004), and no condition by maltreatment history interaction (*F*(1, 52) = .14, *p* = .72, *η*_p_^2^ = 0.003). No interaction of condition and maltreatment history with emotion was found either (*F*(2, 104) = 1.50, *p* = .23, *η*_p_^2^ = 0.028).

**Fig. 2:**
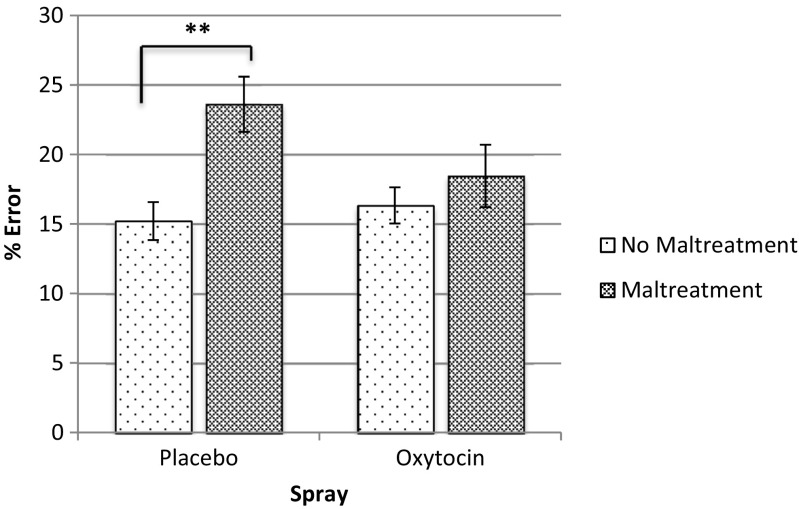
Percentage errors on the working memory task in groups with and without a history of maltreatment in the placebo (*n* = 19 and 9, respectively) and oxytocin condition (*n* = 21 and 7, respectively). Error bars represent standard errors; Double asterisks indicate *p* < .01

When maltreatment history was added as a factor to the RM analyses on percentage of errors, there was again no main effect of condition (*F*(1, 52) = 1.291, *p* = .26, *η*_p_^2^ = 0.024), but there was a significant main effect of maltreatment history (*F*(1, 52) = 8.81, *p* < .005, *η*_p_^2^ = 0.145). Participants who experienced maltreatment were less accurate overall (mean percentage error = 21.35, SD = 1.85) than those who experienced no maltreatment (mean percentage error = 15.80, SD = 0.86). There was furthermore a trend for a condition by maltreatment history interaction (*F*(1, 52) = 3.15, *p* = .082, *η*_p_^2^ = 0.057). Explorative follow-up analyses indicated that participants reporting maltreatment made more errors than those that did not report maltreatment in the placebo condition (*F*(1,26) = 12.79, *p* = .001, *η*_p_^2^ = .33), but not within the oxytocin condition (*F*(1, 26) = .632, *p* = .43, *η*_p_^2^ = 0.024), see Fig. 2. However, within the maltreated group, no significant difference was found between the placebo and the oxytocin condition (*F*(1, 14) = 2.06, *p* = .17, *η*_p_^2^ = 0.13). Lastly, no interaction of condition and maltreatment history with emotion was found (*F*(2, 104) = .53, *p* = .59, *η*_p_^2^ = 0.01).

## Discussion

The goal of this study was to examine whether oxytocin administration would increase interference by emotional information during a working memory task in healthy males. We found no evidence of enhanced distraction by emotional information due to oxytocin on the working memory task. Oxytocin did tend to slow down performance irrespective of distractor valence, but this effect did not reach significance. Furthermore, explorative follow-up analyses indicated that the men reporting a history of childhood maltreatment made more errors in the placebo condition, while they performed similar to the group reporting no history of maltreatment in the oxytocin condition.

As expected, the working memory task showed interference by emotional information. That is, participants responded more slowly after positive and negative distractors compared to neutral distractors and made more errors after negative compared to neutral distractors. This is in line with previous findings showing that this task elicits emotional interference during working memory performance (Krause-Utz et al. 2012; Oei et al. 2010, 2012) and that emotions can interact with cognitive processing (Cromheeke and Mueller 2014). However, in contrast to our expectations, oxytocin did not enhance this emotional interference. While a number of previous studies have indicated that oxytocin administration can lead to augmented processing of emotional information in behavioral tasks, as well as more during more automatic cognitive processes (e.g., Schulze et al. 2011; Van IJzendoorn and Bakermans-Kranenburg 2012; Domes et al. 2013; Tollenaar et al. 2013), others have shown contrasting findings, including reduced attention to negative emotions and null findings (Ellenbogen et al. 2012; Guastella et al. 2009b). The current study does not show enhanced processing of emotions in the context of a working memory task, which again shows the seemingly contextual effects of oxytocin. While these findings may also indicate that oxytocin does not reduce cognitive mental performance in the context of emotional distractors, future studies that examine a wider range of cognitive tasks are recommended to fully elucidate the effects of oxytocin on the interaction between cognitive performance and emotional distractors, including different levels of cognitive load and by including patient groups characterized by emotional dysregulations.

Furthermore, we did find a slight, although non-significant, indication for a slowdown in working memory performance after oxytocin administration. As this reduced performance was independent of distractor type, i.e., in both the neutral and the emotional conditions, it does not appear to depend on the emotional impact or social content of the task. More general impairments in working memory performance after oxytocin administration have previously been shown in animal research (Wirth 2015), and earlier research in humans also suggests that oxytocin can impair memory for (non-social) material (Heinrichs et al. 2004; Herzmann et al. 2012). Wirth (2015) even suggested that oxytocin might exert some of its effects on social behaviors in humans via temporarily inhibiting or impairing working memory by effects on prefrontal brain areas. However, we cannot exclude the possibility that the slower performance after oxytocin administration was due to slightly sedative effects of oxytocin, which have been previously shown (Uvnäs-Moberg et al. 1994). Furthermore, because we used a between-subjects design, this small difference may have been due to baseline group differences.

The most robust finding in the present study was that a history of at least one type of moderate to severe childhood maltreatment was associated with more errors on the task irrespective of distractor valence, while speed was unaffected. This is in line with earlier studies showing associations between childhood trauma and cognitive functioning (DePrince et al. 2009; Majer et al. 2010). However, a previous study with the current working memory paradigm showed more specific emotional interference in the group with a history of childhood maltreatment (Krause-Utz et al. 2012), although this concerned a patient sample. Interestingly, the effect of maltreatment history on task accuracy in the current study was most pronounced in the placebo condition; the maltreated group performed similarly in accuracy to the not maltreated group in the oxytocin condition, suggesting a possible beneficial effect of oxytocin in the maltreated group may exist. However, the sample size of the maltreated group was very small, and performance between the oxytocin and placebo condition within this group did not differ. Therefore, we cannot draw any firm conclusions on the moderating role of maltreatment on the effects of oxytocin administration in the current paradigm. Furthermore, several other studies that have investigated the moderation of oxytocin effects by early life stress have revealed reduced or even negative effects of oxytocin on aspects of social behavior in people with a history of early life stress (Fan et al. 2014; Riem et al. 2013; Van IJzendoorn et al. 2011). Thus, when studying the potential use of oxytocin as an additional therapy for stress-related disorders like anxiety and depression (e.g., Guastella et al. 2009a; Koch et al. 2014; McQuaid et al. 2014), it seems important to examine childhood maltreatment as a possible moderator.

It has been suggested that the amygdala plays a role in the effects of oxytocin on social and emotional task performance (Meyer-Lindenberg et al. 2011; Wigton et al. 2015). Maltreatment has also been associated with enhanced amygdala reactivity in response to emotional information (van Harmelen et al. 2013). However, the oxytocin and maltreatment effects in this study were unrelated to the emotional value of the distractors, and hence, it is unlikely that the current findings are associated with effects on the amygdala. It thus remains unclear whether the current findings are due to general effects on (prefrontal related) working memory processes, or are related to changes in the values of the distractors with potential involvement of the amygdala. Debate continues about the possible different routes by which oxytocin administration may affect behavioral outcomes, i.e., via the enhancement of social salience (Shamay-Tsoory and Abu-Akel 2016), by stimulating approach behaviors (Harari-Dahan and Bernstein 2014), via more general anxiety reducing pathways, or possibly via all of them (Quintana et al. 2015).

Some limitations of the current design should be noted. The current findings are based on a relativity small sample, especially with regard to the maltreatment group as discussed previously. In addition, a between-subjects design was employed, reducing power as well. Within-subjects designs are preferred in pharmacological studies, but our experimental tasks could not all be repeated and precluded the use of a crossover design. We did explorative follow-up analyses of the marginally significant interaction with maltreatment, which should be interpreted with care. Another factor that may be related to the non-significant effects is the timing of the oxytocin administration, which was 80 min before the task was performed. This was due to an EEG paradigm which was employed before the working memory task (Ruissen and de Bruijn 2015). The working memory task was performed last in the study protocol, as the emotional distractors used in the working memory paradigm can be perceived as stressful and could have influenced later tests. However, cognitive and emotional effects of oxytocin have been reported after similar time spans (Olff et al. 2013). With regard to the emotional working memory task, it may have been too easy to elicit strong oxytocin effects, although there was an effect of emotion on both speed and percentage errors. A more challenging version of this task or another type of working memory task could be included in future research. Further, only males were included, and hence, these findings should be replicated in a larger sample, including women as well. With regard to maltreatment as a moderator, future studies should include samples with more variance in amount and types of experienced maltreatment history, or more specifically, participants could be selected for maltreatment history and compared to a matched control group. Controlling the effects of childhood maltreatment for possible confounding by, e.g., social-economic status or general cognitive abilities is also recommended.

### Conclusions

In the context of a working memory task, oxytocin did not enhance distraction by emotional information. Individuals with a maltreatment history made more errors overall, indicating possible problems in working memory for this group. We suggest that effects of oxytocin on working memory should be further explored and could be considered in future studies examining effects of oxytocin on social abilities to further elucidate the pathways by which oxytocin exerts its effects. Furthermore, childhood maltreatment should be considered as a potential moderator.
